# Green carbon science for carbon neutrality

**DOI:** 10.1093/nsr/nwad225

**Published:** 2023-08-25

**Authors:** Zaiku Xie, Buxing Han, Yuhan Sun, Bao-Lian Su, Junlin Yang, Xinhe Bao, Mingyuan He

**Affiliations:** Sinopec Groups, China; Institute of Chemistry, Chinese Academy of Sciences, China; Shanxi Research Institute of Huairou Lab, China; University of Namur, Belgium and Wuhan University of Technology, China; National Natural Science Foundation of China, China; University of Science and Technology of China, China; East China Normal University, China

Nowadays, global warming is becoming more and more serious. The dramatic imagery of global warming frightens people. Carbon is situated at the core of this alarm. It has been scientifically confirmed that global warming is closely related to the increased concentration of CO_2_ in the atmosphere, controlling and reducing carbon emissions has thus become a global consensus. Carbon neutrality by the mid-21st century is essential in order to limit global warming to ≤1.5°C [1]. This means that the atmospheric CO_2_ concentration must be controlled below 450 ppm, which is also specified in the Paris Agreement and signed by 196 countries including the USA, the European Union and China. Besides, it is worth noting that China made a solemn commitment to achieve carbon peaking by 2030 and carbon neutrality by 2060 at the United Nations General Assembly in September 2020, which reflects the responsibility and determination of China to achieve this goal. Up to now, carbon neutrality has become the mainstream direction of global development transformation.

In recent years, world’s major economies, such as the United States, the European Union and China have been continuously releasing national energy development plans, proposing the development of low-carbon technologies and renewable energy to help achieve carbon neutrality. There is much research and work to do, such as improving the efficiency of carbon resource utilization, carbon resource recycling including plastic wastes and biomass transforming, large-scale application and substitution of renewable energy such as solar and wind energy, effective and economical CO_2_ capture, utilization and storage (CCUS), electrification, electrolysis and energy storage by use of green electricity, establishment of a new industrial system based on green hydrogen, etc.

In the above context, to exchange ideas, methods and paradigms related to carbon-neutralization science and technologies, as well as to share or highlight some research progress, we have organized this Special Topic ‘Green Carbon Science—A Scientific Basis for Carbon Neutrality’. Some leading research groups in the fields of chemistry, chemical engineering, and energy across the world have contributed to this Special Topic. This Special Topic features a collection of ideas from academia and industry and includes one highlight, five perspectives, three research articles, three reviews and two interviews. In the article by Mingyuan He and co-workers, the concept of ‘Green carbon science’ was comprehensively elaborated from the aspects of fundamental science and epistemology [2]. Green Carbon Science is a general discipline to develop new scientific knowledge, capability, technology and commercial products based on carbon, thus impacting the way that the world uses and innovates with sustainable carbon materials. They proposed four phases of transformation reactions in the operational functioning of the carbon energy system, as well as four platforms for the development of practical technological solutions focusing on carbon neutrality. In the research article by Xinhe Bao and co-workers, Sr_2_Fe_1.45_Ir_0.05_Mo_0.5_O_6-δ_ perovskite was identified to display a dynamic electrochemical reconstruction feature during CO_2_ electrolysis in solid oxide electrolysis cells (SOECs), which may provide an in-depth understanding of reactive mechanisms of CO_2_ electrolysis [3]. In the research article by Can Li and co-workers, the electron transfer and catalytic reaction processes on monolayer MoS_2_ were investigated at the nanoscale through AFM-SECM mapping, which can help to clarify the function of the applied voltage in the electrocatalytic reaction [4]. In the research highlight, Bao-Lian Su introduces the latest development in the field of photocatalysis concerning integrated multiple techniques to detect the photo induced charge transfer in photocatalytic nanoparticles for uncovering the microscopic mechanism of the photocatalytic hydrogen production process [5]. In the five perspectives, Guangwen Xu and co-workers discussed opportunities, challenges and potential for engineering thermochemistry toward carbon neutralization [6]; Zaiku Xie and co-workers proposed the establishment of a hydrogen based industrial system in order to accelerate the transition to a low-carbon future [7]; Suojiang Zhang and co-workers took three typical products (ammonia, syngas and ethylene) as examples to shed light on how chemical processes are re-engineered by green hydrogen and green electricity coupling with renewable feedstock materials [8]; Qingli Qian and Buxing Han gave a brief overview on the progress of transformation of CO_2_ and H_2_ to C_2+_ chemicals and fuels [9]; Wen-Juan Zhou and Pascal Metivier commented from an industrial point of view impacts along the value chain by a holistic approach, and provided two examples of how the Solvay company tackled sustainability through catalysis science in the specialty chemical industry [10]. In the three review articles, Nikolai Nesterenko, Svetlana Mintova and co-workers outlined potential options for how a methane-to-chemicals process could support decarbonization of the downstream industry [11]; Zhongmin Liu and co-workers discussed the cavity-controlled principle for methanol conversion over zeolite catalysts [12]; Mingfeng Li and co-workers reviewed the chemical recycling process of polyolefin wastes, including moderate pretreatment, pyrolysis, gasification, and refining of pyrolysis oil for olefins, and brief life cycle assessment (LCA) applications that were used to address environmental footprints [13]. Finally, NSR assembled two interviews with academicians Jing-Hai Li and Hou-Liang Dai, two representative scientists from the scientific and industrial circles respectively, on the topic of ‘the future of carbon neutrality technology’ [14,15].

We thank all the authors and editorial staff for their efforts on making this Special Topic possible. Carbon neutrality is an ideal goal. However, the path to achieving it is full of challenges, uncertainties and opportunities, which needs significant progress and breakthroughs on green carbon science. This Special Topic attempts to throw a sprat to catch a whale, hoping to attract the attention of both the scientific and industrial communities in order to engage in more and deeper research in the future.

***Conflict of interest statement**.* None declared.
